# Will an outbreak exceed available resources for control? Estimating the risk from invading pathogens using practical definitions of a severe epidemic

**DOI:** 10.1098/rsif.2020.0690

**Published:** 2020-11-11

**Authors:** R. N. Thompson, C. A. Gilligan, N. J. Cunniffe

**Affiliations:** 1 Mathematical Institute, University of Oxford, Oxford, UK; 2 Christ Church, University of Oxford, Oxford, UK; 3 Department of Plant Sciences, University of Cambridge, Cambridge, UK

**Keywords:** mathematical modelling, infectious disease epidemiology, major epidemic, severe epidemic, forecasting

## Abstract

Forecasting whether or not initial reports of disease will be followed by a severe epidemic is an important component of disease management. Standard epidemic risk estimates involve assuming that infections occur according to a branching process and correspond to the probability that the outbreak persists beyond the initial stochastic phase. However, an alternative assessment is to predict whether or not initial cases will lead to a severe epidemic in which available control resources are exceeded. We show how this risk can be estimated by considering three practically relevant potential definitions of a severe epidemic; namely, an outbreak in which: (i) a large number of hosts are infected simultaneously; (ii) a large total number of infections occur; and (iii) the pathogen remains in the population for a long period. We show that the probability of a severe epidemic under these definitions often coincides with the standard branching process estimate for the major epidemic probability. However, these practically relevant risk assessments can also be different from the major epidemic probability, as well as from each other. This holds in different epidemiological systems, highlighting that careful consideration of how to classify a severe epidemic is vital for accurate epidemic risk quantification.

## 1. Introduction

Infectious disease epidemics in populations of humans, animals and plants represent a recurring risk worldwide [1–7]. An important aim for policy-makers near the start of an outbreak is to assess the risk posed by the invading pathogen, including whether initial cases will lead to a major epidemic or whether the pathogen will die out rapidly instead [8,9]. An important practical consequence is that, if an outbreak is likely simply to fade out, then costly interventions such as vaccination [10,11], culling/felling/roguing of plants or agricultural animals [12–18] and workplace or school closure [19] may be unnecessary [20].

There is a well-known estimate for the probability of a major epidemic when a pathogen is newly arrived in a host population, which in its simplest form is given by

1.1 $$\text{Prob}(\text{major epidemic})=\;1-{\left(\frac{1}{R_{0}}\right)}^{I(0)},$$

in which *R*_0_ is the basic reproduction number of the pathogen and *I*(0) is the number of individuals that are currently infected. The estimate in equation (1.1) applies to a wide range of models, including the commonly used stochastic susceptible–infected–susceptible (SIS) and susceptible–infected–removed (SIR) models [21]. It is derived by assuming that infections occur according to a branching process (see Methods). For the commonly used stochastic susceptible–exposed–infectious–removed model, the exponent in equation (1.1) would change from *I*(0) to *E*(0) + *I*(0) [9]. More sophisticated estimates based on branching process approximations can be derived for models including additional epidemiological detail, such as more complex population structure [22–24] and/or infectious periods that are not exponentially distributed [25,26].

The quantity in equation (1.1), and particularly the version in which *I*(0) = 1, is used extensively in the epidemiological modelling literature [8,9,21,26–35]. It is increasingly used in real-time during emerging outbreaks. For example, it was used during the 2014–2016 epidemic of Ebola virus disease in West Africa to estimate the chance that, if the virus arrived in Nigeria, sustained transmission would follow in that country [30]. It was considered in the context of flare-ups in new locations for the 2018–2020 Ebola epidemic in the Democratic Republic of the Congo [26]. Branching process models were also used at the start of the COVID-19 pandemic before cases were detected outside China to assess the risk of epidemics elsewhere [36,37], including the application of equation (1.1) [36].

**Figure 1.**
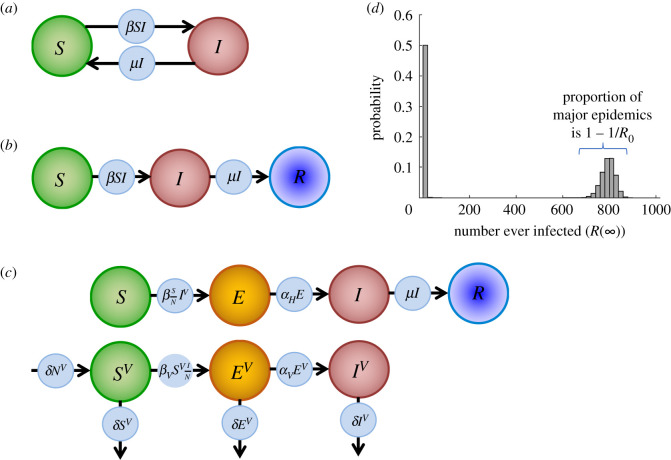
Schematic diagrams illustrating the population structures for the different models considered, and an example distribution of final sizes for the stochastic SIR model. (*a*) The SIS model. (*b*) The SIR model. (*c*) The host–vector model of Zika virus transmission. (*d*) Distribution of final sizes in the stochastic SIR model, with population size *N* = 1000, *R*_0_ = 2, *I*(0) = 1 and the rest of the population susceptible initially. The *x*-axis has been split into bars of width 20 (so that, for example, the first bar corresponds to the probability that between 1 and 20 individuals are ever infected).

However, while the major epidemic probability in equation (1.1) is useful to assess whether or not an outbreak is likely to persist beyond the initial stochastic phase, becoming a major epidemic does not guarantee that the outbreak will overwhelm available control resources. Over many outbreaks under identical conditions, if the population size is large and *R*_0_ is much greater than one, then the distribution of possible epidemic sizes is bimodal according to simple epidemic models such as the stochastic SIR model (figure 1*d*—see also [38–42]). In other words, the final size of any single outbreak is almost always in one of two possible ranges. For example, in figure 1*d*, virtually all outbreaks either lead to 1–20 hosts ever infected or to 700–860 hosts ever infected, where the precise ranges depend on the population size and the value of *R*_0_. The estimate for the probability of a major epidemic in equation (1.1) corresponds approximately to the proportion of outbreaks that have a final size in the higher of these ranges. The outbreaks within the higher range, however, do not necessarily represent outbreaks in which available control resources are exceeded. For practical assessments of the threat from an invading pathogen, it would often therefore be appropriate for the notion of a severe epidemic to be grounded in consequences for disease control, depending on the specific system and outbreak under consideration.

Here, we assess whether or not outbreaks are likely to develop into severe epidemics according to three possible metrics that might be practically relevant in different outbreak scenarios. Specifically, these are:

*Concurrent size*. In this assessment, a severe epidemic is an outbreak in which the number of individuals infected simultaneously exceeds the capacity for treatment.
*Total infections*. In this assessment, a severe epidemic is an outbreak in which the total number of infections exceeds the number of available treatments.
*Duration*. In this assessment, a severe epidemic is an outbreak that is not contained quickly and therefore persists for an unacceptably long period.

We compare the probability of a severe epidemic under each of these definitions, as well as calculate the branching process estimate for probability of a major epidemic (hereafter, we differentiate between the ‘probability of a severe epidemic’ calculated using one of the metrics above and the ‘probability of a major epidemic’ calculated by assuming that infections occur according to a branching process). In our main analyses, as examples we consider three stochastic epidemiological models that are representative of different host responses to infection and capture different routes of transmission. Specifically, we consider the SIS model, the SIR model and a host–vector model parametrized for Zika virus transmission. For the SIS and SIR models, the probability of a major epidemic corresponds to equation (1.1), and in the case of Zika virus the probability of a major epidemic is given by an adapted version of equation (1.1) that accounts for transmission between hosts by vectors (we present an approach for deriving these well-known formulae; see Methods and electronic supplementary material, texts S1 and S2).

To motivate our analyses, we note that estimation of the risk of the outbreak going on to have a large concurrent size, a large total number of infections or a long duration might be the appropriate risk assessment in different scenarios. For example, it might be natural to assume that, if the number of individuals infected at any time always remains below the capacity for treatment, then the outbreak is not severe since medical care is available for all individuals who require treatment. Indeed, in the ongoing COVID-19 pandemic, one of the main aims of interventions in the UK has been to ensure that the number of individuals requiring intensive care unit beds remains below the total number of beds available [43–45]. More generally, the threshold capacity might derive from the number of available beds in hospitals or treatment units [46,47], or the availability of care workers [48]. This motivates consideration of the ‘concurrent size’ metric above.

However, assessing the outbreak risk based on numbers of hosts infected simultaneously will not always be appropriate. Policymakers often have to make decisions concerning how much treatment to stockpile; if all cases must be treated, this corresponds to the total number of infections during the outbreak. For example, in response to growing awareness of the threat of an influenza pandemic, between 2006 and 2013 policymakers in the UK stockpiled around 40 million units of antivirals at a cost of £424 million. This led to severe criticism when only 2.4 million units were needed, the majority of which were used during the 2009 H1N1 influenza pandemic [49]. Another possible risk assessment is therefore whether or not the total number of infections will exceed a critical value (the ‘total infections’ metric above). This critical value might be set by the stock of available treatments for use during the outbreak.

Finally, we consider a third possible risk assessment (using the ‘duration’ metric). In this scenario, we evaluate whether or not an outbreak is likely to persist for an unacceptably long period. An outbreak that fades out quickly may escape public attention. Even if an outbreak leads to a significant number of hosts infected, if it ends relatively quickly then it might not be considered a severe epidemic. For example, the first Ebola outbreak in the Democratic Republic of the Congo in 2018 resulted in 53 cases, but was not considered a severe epidemic due to its fast containment, leading to commendation of the success of public health measures [50]. Consequently, an outbreak might only be classified as a severe epidemic if it persists for a threshold length of time.

An outbreak might be classified as a severe epidemic according to one of the metrics above, yet not be a severe epidemic if another metric is used. In 1665–1666, plague affected the village of Eyam in the UK, which famously isolated itself via a self-imposed quarantine [51,52]. The outbreak in the village was long-running, and a large number of individuals were killed (most reports suggest 250–260 out of a total of 350 in the village died, although there is some uncertainty particularly regarding the size of the at-risk population [53]). However, model fits suggest that a maximum of only around 30 people were ever infected simultaneously [54–56]. As a result, this epidemic might have been classified as severe according to the ‘total infections’ and ‘duration’ metrics, yet not the ‘concurrent size’ metric, depending on the precise values of the thresholds set in each case. This highlights the need to consider the appropriate metric for defining severe epidemics in the particular ongoing outbreak under consideration in order to perform the most practically relevant risk assessment.

A large body of theoretical work exists relating to the metrics for defining severe epidemics that we consider. For example, for the stochastic SIS and SIR models, probability distributions for the maximum number of individuals infected concurrently prior to epidemic extinction have been derived previously [57–59]. For *R*_0_ significantly greater than one, a related quantity (the quasi-stationary distribution—the distribution of the number of infected individuals in the long phase of the epidemic prior to extinction) has been studied in detail for models in which the pathogen persists long-term, including the stochastic SIS model [60–65] and the stochastic SIR model with births and deaths [66]. Analytic expressions and approximations have been found for the total number of infections over the course of an outbreak for a range of epidemic models [25,41,59,67–69] and methods exist for calculating probability distributions describing the possible final sizes of a stochastic epidemic (for a review of approaches for the stochastic SIR model, see [59]). The duration of an epidemic has also been well-studied [60,70–75], as has the duration of the initial stochastic phase of outbreaks that go on to become major epidemics [76].

However, previously developed mathematical theory is not the focus of our analyses. Instead, the novelty of the research that we present is to compare assessments of the risk from invading pathogens evaluated in different ways. We demonstrate the general principle that the precise definition of a severe epidemic (i.e. the metric chosen to define a severe epidemic, or the choice to use the standard branching process estimate for the major epidemic probability) can affect risk assessments whenever a pathogen arrives in a new host population. The probabilities that an outbreak has a large concurrent size, a large total number of infections or a long duration may not coincide and depend on precisely which values of the relevant thresholds are set. These probabilities may or may not match the major epidemic probability assessed in the standard way. Careful consideration of precisely how a severe epidemic is classified is therefore necessary whenever the risk from an invading pathogen is estimated at the beginning of an emerging outbreak. Only once the notion of a severe epidemic has been formally defined—based on criteria of practical relevance for the specific outbreak and setting under consideration—can this risk be properly assessed.

## 2. Methods

We present the results of five analyses in the main text. In the first three, we consider the stochastic SIS, SIR and Zika host–vector models, and assess the risk that a single initial case will lead to an outbreak with a large ‘concurrent size’. Our final two main analyses focus on the stochastic SIS model. We calculate the probability of an outbreak going on to exceed a pre-specified total number of infections (the ‘total infections’ metric) or time (the ‘duration’ metric).

Here, we describe the epidemiological models that we use, the branching process estimate of the major epidemic probability for each model, and calculation of the probability of a severe epidemic under the ‘concurrent size’ metric for each of the models considered. We then explain how the probability of a severe epidemic under the other practically relevant metrics can be obtained for the SIS model, although our methodology generalizes immediately to any model for which a method of stochastic simulation is available.

### 2.1. Epidemiological models

#### 2.1.1. Susceptible–infected–susceptible model

According to the SIS model, at any time each individual in the population is classified to be either (*S*)usceptible to or (*I*)nfected by the pathogen. The deterministic SIS model is given by

2.1 $$\frac{\text{d}S}{\text{d}t}=-\beta IS+\mu I,\;\frac{\text{d}I}{\text{d}t}=\beta IS-\mu I,$$

where *β* represents the infection rate between each susceptible–infected pair and *μ* is the rate at which each infected host recovers and becomes susceptible again. We use the analogous stochastic model in most of our analyses, where the net rate at which any epidemiological event occurs is *βIS* + *μI*. At any time prior to the end of the outbreak, the probability that this next event is an infection is βIS/(βIS+μI) and the probability that the next event is a recovery is μI/(βIS+μI).

In this model, if the total population size is *S* + *I* = *N*, the basic reproduction number is given by $R_{0}=\beta N/\mu .$

#### 2.1.2. Susceptible–infected–removed model

Under the SIR model, at any time each individual in the population is classified according to whether they are (*S*)usceptible to infection, (*I*)nfected, or (*R*)emoved and no longer spreading the pathogen or available for infection. The deterministic SIR model is given by

2.2 $$\frac{\text{d}S}{\text{d}t}=-\beta IS,\;\frac{\text{d}I}{\text{d}t}=\beta IS-\mu I,\;\frac{\text{d}R}{\text{d}t}=\mu I,$$

in which *β* again governs the infection rate and *μ* is the removal rate. In the analogous stochastic model, the net rate at which any epidemiological event occurs is still *βIS* + *μI*, and the probability that the next event is an infection event is similarly unchanged at βIS/(βIS+μI). However, the other possible next event is a removal, which occurs with probability μI/(βIS+μI). The basic reproduction number is again $R_{0}=\beta N/\mu ,$ where in this case *S* + *I* + *R* = *N*.

#### 2.1.3. Zika transmission model

We consider the transmission of Zika virus according to a host–vector model [77], which we chose to demonstrate how the probability of a severe epidemic can be calculated in a relatively complex epidemiological setting. In the model, the numbers of the *N* hosts that are (*S*)usceptible, (*E*)xposed, (*I*)nfectious and (*R*)emoved are tracked, as well as the numbers of the *N^V^* vectors that are (*S*^V^)usceptible, (*E*^V^)xposed and (*I*^V^)nfectious. We adapt the version of the model as presented by Kucharski *et al.* [77] slightly to a more standard formulation in which all transmission terms are proportional to the relevant number of vectors and density of hosts. The deterministic version of this model is then

2.3 $$\begin{array}{rl} \frac{\text{d}S}{\text{d}t}= & -\beta I^{V}\frac{S}{N},\;\frac{\text{d}E}{\text{d}t}=\beta I^{V}\frac{S}{N}-\alpha_{H}E,\;\frac{\text{d}I}{\text{d}t}=\alpha_{H}E-\mu I,\;\frac{\text{d}R}{\text{d}t}=\mu I,\\ \frac{\text{d}S^{V}}{\text{d}t}= & \delta N^{V}-\beta_{V}S^{V}\frac{I}{N}-\delta S^{V},\;\frac{\text{d}E^{V}}{\text{d}t}=\beta_{V}S^{V}\frac{I}{N}-(\delta +\alpha_{V})E^{V}\\ \text{and}\;\frac{\text{d}I^{V}}{\text{d}t}= & \alpha_{V}E^{V}-\delta I^{V}. \end{array}\}$$

The parameters *β* and *β_V_* govern the rates at which infectious vectors infect susceptible hosts and susceptible vectors acquire the pathogen from infectious hosts, respectively. The mean latent period of infections in hosts is 1/*α*_*H*_, and exposed vectors become infectious at rate *α*_*V*_. The parameter *μ* is the rate of removal of infectious hosts, and *δ* describes the death rate of every vector. In the analogous stochastic model, the expected number of infected human hosts arising from a single infected human (accounting for human–vector–human transmission) in an otherwise entirely susceptible population of humans and vectors is given by

$$R_{0}^{HV}\times \rho^{E^{V}\to \;I^{V}}\times R_{0}^{VH}=\frac{\beta_{V}\alpha_{V}\beta N^{V}}{\mu (\delta +\alpha_{V})\delta N},$$

where $R_{0}^{HV}=\;(1/\mu )\;(\beta_{V}N^{V}/N)$ is the expected number of vectors infected (and going on to enter the exposed class) by a single infectious human, $\rho^{E^{V}\to \;I^{V}}=\alpha_{V}/(\delta +\alpha_{V})$ is the proportion of exposed vectors that become infectious and $R_{0}^{VH}=\beta /\delta$ is the expected number of humans infected by a single infectious vector.

The basic reproduction number is given by $R_{0}=\sqrt{\left(\beta_{V}\alpha_{V}\beta N^{V})/(\mu (\delta +\alpha_{V})\delta N\right)},$ where the square root accounts for the fact that it takes two generations for infected humans to generate new infections, since new infections require host–vector–host transmission [78,79]. We note that in some studies, e.g. [77], the square root is omitted from the definition of *R*_0_. In contrast to the expression calculated by Kucharski *et al*. [77], to facilitate simulation of the stochastic model we consider the total number of vectors, *N^V^*, rather than the density.

### 2.2. Probability of a major epidemic (branching process estimate)

#### 2.2.1. Standard estimate (stochastic SIS/SIR models)

The commonly used estimate for the major epidemic probability when a pathogen first arrives in a host population [8,9,21,27,29–36] can be derived by assuming that infections occur according to a branching process, making the assumptions that the susceptible population is large and that infection lineages arising from different infected hosts are independent. When a single infected host arrives in an otherwise susceptible population, the branching process estimate for the major epidemic probability is given by

$$\text{Prob}(\text{major epidemic})\approx \{\begin{array}{cc} 0 & \;\text{for}\;R_{0}\leq 1,\\ 1-\frac{1}{R_{0}} & \;\text{for}\;R_{0}>1. \end{array}$$

This expression is derived in electronic supplementary material, text S1.

If instead there are *I*(0) infected individuals initially rather than one, then for no major epidemic to occur, it is necessary for each initial infection lineage to die out, leading to the approximation given in equation (1.1) whenever *R*_0_ > 1.

#### 2.2.2. Standard estimate (Zika transmission model)

The branching process estimate for the major epidemic probability starting from a single infected host for the stochastic Zika transmission model is derived in electronic supplementary material, text S2, and is given by

2.4 $$\text{Prob}(\text{major }\mathrm{epidemic})\approx \{\begin{array}{cc} 0 & \;\text{for}\;R_{0}\leq 1,\\ \frac{{\left(R_{0}\right)}^{2}-1}{{\left(R_{0}\right)}^{2}+R_{0}^{VH}} & \;\text{for}\;R_{0}>1. \end{array}$$

In this expression, $R_{0}^{VH}$ is the expected number of humans infected by a single infectious vector in an otherwise entirely susceptible population of humans and vectors.

### 2.3. Probability of a severe epidemic (‘concurrent size’ metric)

Under the ‘concurrent size’ metric, we define a major epidemic to be an outbreak in which the maximum number of individuals infected simultaneously is above a threshold value, which we denote by *M*. The value of *M* of relevance in practical applications might be set by the capacity for treatment.

#### 2.3.1. Stochastic susceptible–infected–susceptible model

Under the stochastic SIS model, the probability that the number of individuals infected simultaneously is at least *M* at some time prior to epidemic extinction can be calculated analytically [57]. This is advantageous since approximating this quantity using model simulations can be time consuming given that outbreaks under the SIS model can persist for long periods. Specifically, as derived in electronic supplementary material, text S3,

2.5 $$\text{Prob}(\text{severe}\;\text{epidemic})=\{\begin{array}{ccc} 0 & \;\text{for} & I(0)=0,\\ \frac{1}{1+\sum_{I=1}^{M-1}A_{I}} & \;\text{for} & I(0)=1,\\ \frac{1+\sum_{I=1}^{I(0)-1}A_{I}}{1+\sum_{I=1}^{M-1}A_{I}} & \;\text{for} & 1<I(0)<M,\\ 1 & \;\text{for} & I(0)\geq M, \end{array}$$

in which

$$A_{I}=\frac{{\left(N/R_{0}\right)}^{I}}{I!\left(\begin{array}{c} N-1\\ I \end{array}\right)}.$$

#### 2.3.2. Stochastic susceptible–infected–removed model

For the stochastic SIR model, the probability that the maximum number infected simultaneously is at least *M* starting from any state (*I*,*R*) is calculated using an iterative approach [58,59]. Denoting the probability of a severe epidemic starting from state (*I*,*R*) by *p_I_*_,*R*_, then conditioning on the next event gives

$$p_{I,R}=\frac{\beta I(N-I-R)}{\beta I(N-I-R)+\mu I}p_{I+1,R}+\frac{\mu I}{\beta I(N-I-R)+\mu I}p_{I-1,R+1}.$$

This system can be solved with boundary conditions *p*_0,*R*_ = 0, *p_I_*_,*N*−_*_M_*_+1_ = 0 and *p_M_*_,*R*_ = 1. Doing so does not require this system of equations to be solved simultaneously. Instead, the value of *p_I_*_,*R*_ is deduced for the following states (in order): (*I*,*R*) = (*M* − 1, *N* − *M*), (*M* − 2, *N* − *M*), …, (1, *N* − *M*), (*M* − 1, *N* − *M* − 1), …, (1, *N* − *M* − 1), … (*M* − 1, 0), …, (1, 0). For a schematic showing the order in which these probabilities are deduced, see electronic supplementary material, figure S1.

#### 2.3.3. Zika transmission model

For the Zika transmission model, the probability of a severe epidemic with the ‘concurrent size’ metric is approximated using model simulations. The model is simulated 10 000 times using the Gillespie direct method [80]. The probability of a severe epidemic is then approximated by calculating the proportion of simulations in which the number of infected human hosts is at least *M* at any time during the simulation.

### 2.4. Probability of a severe epidemic (‘total infections’ and ‘duration’ metrics)

We also consider the probability of a severe epidemic according to the stochastic SIS model for the ‘total infections’ and ‘duration’ metrics. Specifically, we estimate the probability that at least *F* infections occur over the course of the outbreak (prior to outbreak extinction), and the probability that the outbreak persists for at least *T* days.

We approximate these quantities by simulating the model 10 000 times using the Gillespie direct method [80] and recording separately the proportion of simulations in which there are at least *F* infections or in which the duration is at least *T* days. Each simulation is stopped when either of the following two criteria are satisfied: (i) the simulated outbreak has gone extinct (*I* = 0), or; (ii) both the number of infections has reached the maximum value of *F* considered (*F* = 2000) and the duration has reached the maximum value of *T* considered (*T* = 6000).

## 3. Results

To begin to explore outbreak dynamics under the SIS, SIR and Zika transmission models, we first numerically solved the deterministic models given by the systems of equations (2.1), (2.2) and (2.3) with *R*_0_ = 1.5 in each case (electronic supplementary material, figure S2). For the parameter values considered, the deterministic SIS model predicts the largest number of individuals infected simultaneously as well as the most infections in total. Epidemics persisted forever (i.e. *I* remained larger than zero) under all three models, although the number of infected hosts tended to zero under the SIR and Zika transmission models.

**Figure 2.**
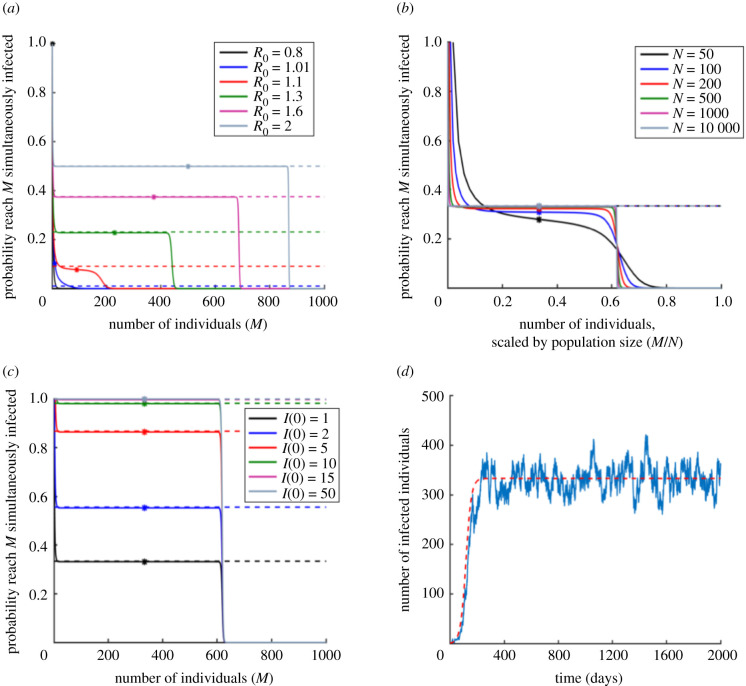
Probability of a severe epidemic under the SIS model, where a severe epidemic is defined as an outbreak in which at least *M* individuals are infected simultaneously at some time during the outbreak (‘concurrent size’ metric). (*a*) Dependence on *R*_0_. Solid lines represent the probability of a severe epidemic (system of equations (2.5)), dotted lines represent the branching process estimate for the major epidemic probability (equation (1.1)) and dots show the maximum number simultaneously infected in the analogous deterministic models (calculated analytically as shown in electronic supplementary material, text S4). *R*_0_ is varied by changing the value of *β*. (*b*) Equivalent to (*a*), but showing dependence on the population size, *N*. (*c*) Equivalent to (*a*), but showing dependence on the initial number of infected individuals, *I*(0). (*d*) Single simulation of the stochastic SIS model (blue) and numerical solution of the deterministic SIS model (red dotted). The value of *I* in the stochastic simulation will continue to fluctuate about the deterministic value until *I* reaches 0. Parameter values (except where stated): *N* = 1000, *R*_0_ = 1.5, *I*(0) = 1 and the remainder of the population susceptible initially. In panel (*d*), *β* = 0.00015 per day and 1/*μ* = 10 d.

**Figure 3.**
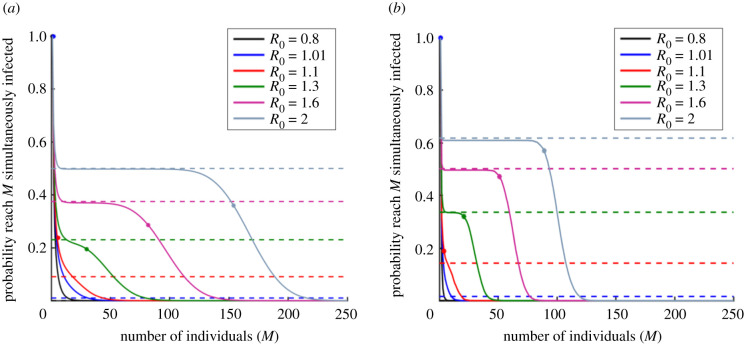
Probability of a severe epidemic under the SIR and Zika virus transmission models, where a severe epidemic is defined as an outbreak in which at least *M* individuals are infected simultaneously at some time during the outbreak (‘concurrent size’ metric). (*a*) SIR model. Solid lines represent the true probability of a severe epidemic calculated using the iterative method described in Methods, dotted lines represent the branching process estimate of the probability of a major epidemic (equation (1.1)) and dots show the maximum number simultaneously infected in the analogous deterministic model (calculated analytically for the SIR model as shown in electronic supplementary material, text S4). *R*_0_ is varied by changing the value of *β*. (*b*) Equivalent to (*a*), but for the Zika virus transmission model (where *M* refers to the number of simultaneously infected hosts). For the Zika transmission model, the probability of a severe epidemic is calculated by simulation, and the branching process estimate of the major epidemic probability is given by equation (2.4). The maximum number simultaneously infected is found for the deterministic Zika virus transmission model by numerically solving the model. For both models, *N* = 1000. Other parameters for the Zika virus transmission model: *N^V^* = 10 000, 1/*α_V_* = 10.5 d, 1/*α_H_* = 5.9 d, 1/*μ* = 5 d, 1/*δ* = 7.8 d, *β_V_* = 0.22 per day [77]. Initial conditions for both models comprise a single infected host, with all other individuals (for the Zika transmission model, hosts and vectors) susceptible.

**Figure 4.**
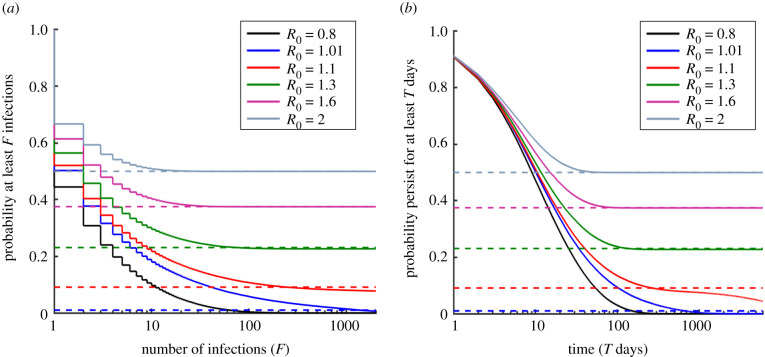
Probability of a severe epidemic under the SIS model, for different definitions of a severe epidemic. (*a*) A severe epidemic is defined as an outbreak in which at least *F* infections occur (‘total infections’ metric). (*b*) A severe epidemic is defined as an outbreak that persists for at least *T* days (‘duration’ metric). Solid lines represent the probability of a severe epidemic assessed via repeated simulation of the stochastic model, and dotted lines represent the branching process estimate for the probability of a major epidemic (equation (1.1)). The *x*-axis is shown on a log-scale, for *F* between 1 and 2000 (*a*) and *T* between 1 and 6000 (*b*). The step function in panel (*a*) reflects the fact that the total number of infections can only take integer values. Results of the deterministic model are not included in the figure, since under the deterministic SIS model epidemics persist indefinitely and generate an infinite number of infections whenever *R*_0_ > 1. Parameter values: *N* = 1000, *I*(0) = 1 and the remainder of the population susceptible initially. In both panels, *R*_0_ is varied by changing the value of *β*. In panel (*b*), 1/*μ* = 10 d.

However, our main focus is assessing the risk from an invading pathogen according to the more realistic stochastic models. In the following sections, first we calculate the probability of a severe epidemic for the stochastic SIS model using the ‘concurrent size’ metric. We then consider the other epidemiological models, as well as the other metrics defining a severe epidemic. In each case, the probability of a severe epidemic for the particular epidemiological model-severe epidemic metric pair under consideration is compared with the branching process approximation to the probability of a major epidemic for that model. The rationale for this comparison is that both quantities represent a possible way to assess the risk from an invading pathogen. Results are shown in figures 2–4 and summarized in electronic supplementary material, tables S1 and S2. A chart outlining the model-severe epidemic metric pairs considered in each figure is shown in electronic supplementary material, figure S3.

### 3.1. The probability of a severe epidemic

We calculated the probability of a severe epidemic according to the stochastic SIS model under the ‘concurrent size’ metric for a severe epidemic—i.e. an outbreak in which the number of individuals infected simultaneously is at least a pre-specified threshold number (*M*) at some time during the outbreak. In this case, as described in Methods, it is possible to calculate the probability of a severe epidemic analytically.

We show the probability of a severe epidemic for a range of values of the threshold *M* in figure 2*a*. For *R*_0_ larger than but not close to one, the probability of a severe epidemic was approximated closely by the standard branching process estimate for the probability of a major epidemic for many values of the threshold, *M*. When, however, *R*_0_ was close to one, the standard estimate corresponded to a single choice of *M* (see e.g. blue and red lines in figure 2*a*, where the solid line is close to the corresponding dotted line in only one place, i.e. for a single value of *M*). The parameter regime in which *R*_0_ is close to one is important in many epidemiological systems since the aim of pre-emptive control strategies is often to reduce *R*_0_ below one (or, when an outbreak has started, to reduce the time-varying or effective reproduction number below one [81–84]).

In large host populations, the probability of a severe epidemic as a function of *M* took the form of a step function (figure 2*b*). If the pathogen successfully invaded the population, then the proportion of the population infected simultaneously (rather than number of individuals infected simultaneously) would definitely reach a specific maximum value which is determined by *R*_0_. For example, for outbreaks with *R*_0_ = 1.5, the pathogen will invade the population with probability 0.33 (i.e. the probability of a major epidemic), and, if this occurs, then around two-thirds of the population will be infected simultaneously at some time during the epidemic. In this case (*R*_0_ = 1.5), conditional on invasion, the maximum value of *I* prior to epidemic extinction in the stochastic SIS model corresponds to approximately double the maximum value of *I* in the deterministic model, reflecting the roughly symmetric fluctuation of *I* in the SIS model about the deterministic endemic equilibrium (figure 2*d*). In figure 2*b*, when *N* = 10 000, this approximation gives a maximum proportion of the population simultaneously infected of 0.67, when the true value is 0.62.

### 3.2. Different epidemiological models

We considered the probability that the maximum number of individuals infected simultaneously is at least a pre-specified threshold (i.e. a severe epidemic occurs, using the ‘concurrent size’ metric) under the SIR and Zika virus transmission models. For the stochastic SIR model, we used an iterative method to calculate this probability as described in Methods. For the stochastic Zika virus transmission model, we simulated the model in a population of *N* = 1000 human hosts and *N*^V^ = 10 000 vectors using the Gillespie direct algorithm [80] with parameter values from Kucharski *et al.* [77], and calculated the proportion of simulations in which a severe epidemic occurred—see caption of figure 3. The value of *R*_0_ was then varied in figure 3*b* by altering the parameter *β* that governs the rate at which infected vectors infect susceptible human hosts.

Under the stochastic SIR and Zika virus transmission models, for *R*_0_ larger than and not close to one, the maximum number of simultaneously infected individuals whenever the pathogen invaded the host population was typically smaller than under the SIS model (cf. electronic supplementary material, figure S2). Nonetheless, we found qualitatively similar behaviour in these cases—the probability of a severe epidemic was similar to the major epidemic probability approximated using a branching process for a wide range of values of the severe epidemic threshold when *R*_0_ was high (figure 3). However, even if that is the case, the practically relevant value of the severe epidemic threshold (e.g. the number of available hospital beds) may mean that the severe epidemic probability does not match the major epidemic probability. For example, in figure 3*a*, if *R*_0_ = 2 and 250 beds are available, the probability of a severe epidemic under the ‘concurrent size’ definition is 0 (solid grey line in figure 3*a*), yet the branching process estimate for the probability of a major epidemic is 0.5 (dotted grey line in figure 3*a*).

### 3.3. Alternative definitions of a severe epidemic

For the stochastic SIS model, we then calculated the probability of a severe epidemic using different metrics to define a severe epidemic—specifically, outbreaks in which there are at least *F* infection events (the ‘total infections’ metric—figure 4*a*) or outbreaks that persist for at least *T* days (the ‘duration’ metric—figure 4*b*).

In the stochastic SIS model, if the pathogen invaded the host population then it tended to persist for long periods. Consequently, the probability of a severe epidemic using the ‘total infections’ or ‘duration’ metrics is approximately equal to the major epidemic probability for a wide range of values of the severe epidemic thresholds (i.e. values of *F* or *T*) compared with under the ‘concurrent size’ definition. However, even in these cases, for small or very large values of the severe epidemic thresholds, the probability of a severe epidemic does not match the major epidemic probability, particularly when *R*_0_ is larger than but close to one (see e.g. red line in figure 4*b*). The probability of a severe epidemic also may or may not match when different metrics are used to classify a severe epidemic, depending on the precise values of the thresholds set (figures 2*a* and 4). This indicates that the most practically relevant metric and threshold to use, or the choice to use the branching process estimate for the major epidemic probability, should be considered carefully when epidemic risks are assessed during emerging outbreaks.

## 4. Discussion

Evaluating the risk from an invading pathogen early in a potential severe epidemic is vital for planning interventions and determining whether or not current control or treatment resources are sufficient. When a pathogen arrives in a new location, the probability that initial cases will lead to a major epidemic as opposed to fading out as a minor outbreak can be approximated by assuming that infections occur according to a branching process. This probability represents the risk that the outbreak will persist beyond the initial stochastic phase in which case numbers are low. For simple models such as the stochastic SIS and SIR models, this corresponds to the major epidemic probability in equation (1.1). This can be extended to estimate the major epidemic probability using models with additional complexity, as we demonstrated by considering the case of host–vector transmission (see equations (2.3) and (2.4)).

However, the threat from an invading pathogen can also be assessed by estimating the probability that the outbreak will become ‘severe’ and overwhelm available control resources. For example, the probability that the peak prevalence will exceed the capacity of treatment facilities (e.g. the number of hospital beds) can be estimated (figures 2 and 3), as well as the probability that the outbreak will exceed a threshold in the total number of infections or will have a long duration (figure 4). In each case, the probability of a severe epidemic depends on the value of the threshold set (e.g. the exact number of hospital beds) to differentiate severe epidemics from other outbreaks, as well as the metric used to define a severe epidemic. This highlights the need to consider the precise definition of a ‘severe epidemic’ carefully when assessing the risk from an invading pathogen.

When *R*_0_ is much greater than one or when the population size is extremely large, however, the probability of a severe epidemic is constant for a range of values of the threshold differentiating severe epidemics from other outbreaks (see e.g. different values of *M* in figure 2*a*). In these cases, the probability of a severe epidemic will often match between definitions. This is perhaps unsurprising since, for example, an outbreak with a large total number of infections may well also have a large peak prevalence. The probability of a severe epidemic is then approximately equal to the major epidemic probability calculated in the standard way (electronic supplementary material, table S2). However, even in this case, the specific threshold of practical importance in the outbreak may correspond to a different probability of a severe epidemic compared with the probability corresponding to a wide range of threshold values. Consequently, if a policymaker wishes to understand the risk that an invading pathogen will overwhelm available control resources, then this question should be addressed directly by choosing the relevant metric and threshold value carefully.

We considered practical definitions of a severe epidemic that were based on thresholds such as the availability of treatment. A previous study defined severe epidemics according to a threshold in the percentage of the population ever infected, and concluded that epidemiological modellers should report the precise cut-off used to define such epidemics in model simulations [85]. Their conclusion was based on the observation that different thresholds in the percentage of hosts ever infected corresponded to wide variations in the other outputs of model simulations including the number of dead hosts or the time of the epidemic peak. We support this conclusion, and indeed some authors have reported the definition of a severe epidemic they used clearly—for example, Keeling *et al*. [86] differentiate between outbreaks in which less than one-third of the population becomes infected and those in which larger numbers of individuals are ever infected. Similarly, a recent study explored the risk of resurgence of COVID-19 when interventions are removed in different countries [87]. In that study, resurgence was said to have occurred when the number of individuals who are symptomatic infectious reached 100. Other studies have also defined ‘large outbreaks’ as those in which a threshold number of cases is exceeded [88,89]. However, while threshold values were reported clearly in all these studies, we emphasize that the precise type of threshold and the value used should be chosen according to practical relevance in the particular scenario under consideration.

Under the first definition of a severe epidemic that we considered (using the ‘concurrent size’ metric), the probability of a severe epidemic was assessed in the context of the capacity for treatment by estimating whether or not a threshold number of simultaneously infected individuals was likely to be exceeded. This definition may be practically relevant in a range of scenarios. For example, real-time analysis of a diphtheria epidemic in Cox's Bazar district in Bangladesh involved assessing the number of hospital beds that were needed [46]. The number of beds required was approximated in that study by using a model to forecast disease incidence, assuming that 15% of reported cases would require treatment as inpatients with an average hospital stay of 5 days for each case. The number of hospital beds that were already available might have provided a practically relevant severe epidemic threshold. Another example for which this type of threshold might apply is citrus greening disease in Brazil, where a law was introduced stating that a citrus grove must be destroyed if 28% of trees in the grove were infected and symptomatic [90,91]. At a local spatial scale, severe epidemics could therefore be defined as outbreaks in which more than 28% of trees in a grove are infected and symptomatic concurrently. Other examples for which interventions were introduced as soon as a threshold in the number simultaneously infected was reached include the development of the National Chlamydia Screening Programme in the United Kingdom in 2002 in response to the large size of the infected population [92].

However, no single metric for determining whether or not an outbreak is a severe epidemic will be relevant in all situations. We therefore also considered two other definitions of a severe epidemic. In one of these (when the ‘total infections’ metric was used), whether or not an outbreak was classified as a severe epidemic referred to the total number of infection events over the course of the outbreak, rather than the maximum number simultaneously infected. This might correspond to the total number of treatments required, which may be an important threshold if treatments have been stockpiled prior to the outbreak [49]. This definition might also be relevant if, for example, a policymaker has to choose how to deploy resources between two different epidemics. If there are only sufficient resources to contain one outbreak, and both epidemics are equally controllable, then—in the absence of other considerations—it might be preferable to choose to contain the epidemic that is likely to generate more infections. In other real-world scenarios, alternative definitions might be appropriate. We also considered classifying severe epidemics as outbreaks that persist beyond a threshold length of time (using the ‘duration’ metric). Different definitions of a severe epidemic might appear contradictory—for example, treatment can act to reduce the total number of infections yet increase the outbreak duration [93], making a severe epidemic less likely when the ‘total infections’ metric is used but more likely when instead the ‘duration’ metric is used.

Our intention here was to use simple models to demonstrate the principle that different approaches for evaluating the threat from an invading pathogen can lead to very different assessments of risk. As described in the introduction, our research builds on a rich history of analyses that relate to the results obtained here. For example, for the stochastic SIS model, it is well known that the time to extinction varies with *R*_0_ [62]. For *R*_0_ < 1, outbreaks will certainly end quickly. When *R*_0_ > 1, however, if *R*_0_ is increased then the expected duration of the epidemic also increases. For fixed *R*_0_, the expected duration grows exponentially with the population size, *N* [60–62]. An exact analytic expression describing the range of possible durations of a stochastic SIS epidemic has not been found, and so we assessed the probability of a severe epidemic under the ‘duration’ metric using model simulations (figure 4). However, analytic approximations to the expected duration of a major epidemic exist (e.g. [60,75]), and exploring the relationship between these approximations and the probability of a severe epidemic for different values of the threshold under the ‘duration’ metric represents an interesting avenue for further investigation.

Another important extension of the research presented here is to explore the risk of a severe epidemic for outbreaks that do not assume that the population is well-mixed. The field of contact network epidemiology provides a framework in which the risk from an invading pathogen can be explored, accounting for the topology of the underlying network when making epidemiological predictions and planning public health measures. In that context, the probability of a major epidemic can be derived, depending on the transmissibility (*T*_1_) of the pathogen rather than *R*_0_ [94]. The value of *T*_1_ represents the average probability that an infected host will transmit the pathogen to a susceptible individual that they have contact with. Meyers *et al.* [95] investigated the dependence of the major epidemic probability on the degree of the index case, as well as the major epidemic probability for different numbers of initial cases, in the context of SARS. Those authors, as well as Pourbohloul *et al.* [96], explored the effects of different interventions that reduce the numbers of contacts on quantities including the probability of a major epidemic. The framework underlying these models has also been extended to account for the time evolution of outbreaks [76], which is imported if the evaluation of the risk from an invading pathogen is to be linked to the extinction time of the outbreak (as in the ‘duration’ metric that we considered).

Although our approach could be extended for different types of models (such as network models), compartmental models (such as the SIS and SIR models) are commonly used for assessing outbreak risks. Accurate outbreak forecasting using a compartmental model requires the model to be carefully matched to the epidemiology of the host–pathogen system, potentially including within-host dynamics [97,98], asymptomatic transmission [9,99,100] or spread between spatially distinct regions [29,101]. For certain definitions of a severe epidemic, it may be necessary to include bed-ridden or convalescent hosts in the model explicitly. For example, if the definition of a severe epidemic is linked to the availability of beds in treatment centres (as may be the case when the ‘concurrent size’ metric is used), then infected individuals in treatment centres could be included in the model explicitly (for an example in which we consider three different models of an Ebola epidemic with different levels of complexity, see electronic supplementary material, text S5). Other definitions of severe epidemics could be used, potentially considering factors such as access to healthcare; limited healthcare access is a particular challenge in low resource settings [81]. It would also be possible to require multiple criteria to be satisfied for an outbreak to be classified as a severe epidemic. In these more complicated scenarios, analytic calculations of the probability of a severe epidemic might not be possible. Model simulations can then be used to assess the risk from the invading pathogen, as we showed for a host–vector model of Zika virus transmission (figure 3*b*).

We note that practical use of the methods presented here at the start of an emerging outbreak to assess the outbreak risk might require the parameters governing pathogen transmission to be estimated directly from case notification data. A range of methods exist for estimating reproduction numbers in real-time during outbreaks [82,83,102–104], including those designed for estimation in the early stochastic phase [105,106]. Practical use of the approaches that we have developed might also require the wide range of interventions that are introduced in outbreak responses to be integrated into the models explicitly. One way in which control can be included is to consider the effective reproduction number when the pathogen arrives in the system instead of the basic reproduction number, since the effective reproduction number accounts for interventions [26,81–83,107–109]. In that situation, the results that we presented would be unchanged (except that e.g. the lines in figure 2*a* would correspond to different values of the effective reproduction number rather than the basic reproduction number). Temporal changes in interventions once an outbreak is underway have been approximated in epidemiological models by assuming that the values of the parameters governing transmission change during the outbreak, either by assuming that transmissibility changes at single timepoints [110,111] or continuously as the outbreak progresses [112,113]. However, for detailed descriptions of control to be included in estimates of the severe epidemic risk, more complex interventions should be included in model simulations. Since models are often used to test possible control strategies [7,12,20,45,81,114–116], this is a simple extension of the results presented here.

In summary, we have shown that the precise definition of a severe epidemic should be considered carefully in future studies that aim to evaluate the risk when a pathogen first arrives in a host population. Only once a severe epidemic has been defined precisely for the specific outbreak and setting under consideration can the epidemic risk be properly assessed. Providing an explicit demonstration of the consequences of not considering the practically relevant definition in evaluating the risk is the key contribution of this paper.

## Data accessibility

All data are included in the manuscript and electronic supplementary material. Sufficient information and data are provided in and with the manuscript to allow others to replicate all study findings reported in this research article.
